# Assumptions behind scoring source and item memory impact on conclusions about memory: A reply to Kellen and Singmann's comment (2017)

**DOI:** 10.1016/j.cortex.2017.08.011

**Published:** 2017-11

**Authors:** Elisa Cooper, Andrea Greve, Richard N. Henson

**Affiliations:** Medical Research Council Cognition and Brain Sciences Unit, Cambridge, England, United Kingdom

In our recent article in the journal *Cortex* (Cooper, Greve, & Henson, 2017), we examined memory for source and item information using data from two different source monitoring paradigms and six different groups of participants. When comparing standard accuracy analysis and various Multinomial Processing Tree (MPT) models, we found that the type of analysis determined the extent to which item and/or source memory differences were found across groups (healthy young and older groups, an older group with mild memory problems, and individuals with hippocampal lesions). Our main point was methodological: that one could draw different conclusions (e.g., whether ageing or hippocampal lesions affect only source memory, or both source and item memory) depending on the analysis used.

In our paper, we considered two MPT models that differed in their tree structure. In one of the two models – what we called the “Item-Source” model – the parameter representing accurate source memory occurs “downstream” of the parameter representing accurate item memory. This is the only type of tree structure that we have seen considered in the extensive literature of MPT models of source memory (e.g., Batchelder and Riefer, 1990, Bayen et al., 1996, Riefer and Batchelder, 1988), and would seem to correspond to the common assumption that remembering the source in which an item occurred is conditional on remembering the item itself. We contrasted this model with an alternative “Source-Item” model, in which the parameter representing accurate source memory occurs “upstream” of the parameter representing accurate item memory. We likened this to dual-process models of memory (e.g., Yonelinas, 1999), in which two processes operate: one in which the full memory is retrieved (item and source, akin to recollection) and another in which only item memory is retrieved (akin to familiarity). There is considerable evidence from neuropsychology and neuroimaging that these two processes are supported by distinct brain systems [involving hippocampus and perirhinal cortex respectively, (Norman and O'Reilly, 2003, Yonelinas, 2002)]. We appreciate that there is not a one-to-one mapping from such theoretical models to MPT structures, and that yet further tree structures are possible, depending also on how source memory decisions are made. Our point was only to illustrate how two example MPT structures can be associated with different theoretical frameworks.

In their commentary on our paper, Kellen and Singmann (2017) focus on our second paradigm, where we extended the two MPT models to capture additional confidence judgements. They observe that some of the parameters *within* each of our MPT models are not monosemous, i.e., parameters do not have the same meaning when occurring at different points within the tree structure, and show that this is the reason why the models produce different fits to the data. We accept this criticism, and agree that MPT parameters should have the same meaning within a tree, and therefore we could not support one model over the other by virtue of their goodness of fit to the data in our second paradigm. However this issue is irrelevant to our larger claim that parameters have different meanings *across* models, even when they have the same meaning within each model and even when two models fit the data equally well, as we demonstrate below.

Kellen and Singmann unfortunately ignore the points we made with our first paradigm. In this simpler paradigm, the parameters within the Item-Source model and the Source-Item model are monosemous, and the models fit the data equally well. Indeed, we demonstrated the models' equivalence formally in the Supplementary Material of our original paper (www.sciencedirect.com/science/article/pii/S0010945217300047), showing that the parameters of one model can be expressed in terms of the parameters of the other (as Kellen and Singmann also do for our second paradigm). However, this does not change the fact that one will obtain different numerical (and possibly statistical) results from each model when considering these parameters in isolation, and therefore potentially arrive at different conclusions about how conditions or groups differ on each parameter.

Evidence for this can be seen in the individual parameter estimates from our first paradigm, but we also work through an example here. Imagine that the “true” model is the Source-Item model and that hippocampal lesions affect the $D_{s}$ parameter (reflecting source and item memory), but not the $D_{i}$ parameter (reflecting item memory without source memory). Using the equations in the Supplementary Material available from the link above, the corresponding values for the Item-Source model, denoted by ${\tilde{D}}_{s}$ and ${\tilde{D}}_{i}$, would be: ${\tilde{D}}_{i}=1-\left(1-D_{s}\right)\left(1-D_{i}\right)$ and ${\tilde{D}}_{s}=D_{s}/{\tilde{D}}_{i}$

Now if we have two groups of participants denoted by superscripts *C* for Controls and *H* for Hippocampal lesions, we can take example values of $D_{s}^{C}=0.6>D_{s}^{H}=0.4$, and $D_{i}^{C}=D_{i}^{H}=0.8$. If we plug these values into the above equations, we find that ${\tilde{D}}_{s}^{C}=0.6522$ and ${\tilde{D}}_{s}^{H}=0.4545$. These values of the “source memory” parameters from the Item-Source model are lower than those from the Source-Item model (which is fine, because the parameters mean different things), but are at least of the same relative size across groups. However, the values of the “item memory” parameters in the Item-Source model now differ between groups, in that ${\tilde{D}}_{i}^{C}=0.92$ and ${\tilde{D}}_{i}^{H}=0.88$, even though values in the Source-Item model are identical, $D_{i}^{C}=D_{i}^{H}=0.8$. If the meanings of $D_{i}$ and ${\tilde{D}}_{i}$ were equated across the two models, one would falsely conclude that hippocampal lesions affect item memory according to the Item-Source model where the parameter estimates differ, but not the Source-Item model. However, the parameters do *not* have the same meaning across the two models, which is how they can differ: in the Source-Item model, $D_{i}$ refers to the probability of item memory without source memory, whereas in the Item-Source model, ${\tilde{D}}_{i}$ refers to the probability of item memory only (with or without source memory). Nonetheless, even though parameters are not directly comparable across models, the fact remains that one would draw quite different scientific conclusions from testing individual parameters across groups, depending on the model used. Moreover, the choice of model depends on one's *a priori* theory (particularly when models fit the data equally well).

In summary, both we and Kellen and Singmann agree that there is no theory-neutral way of scoring data. This is indeed a claim that has been made many times before (but often forgotten nonetheless). The purpose of our paper was to demonstrate the impact that such different theoretical assumptions have on conclusions drawn from real data, such as the effect of age or hippocampal lesions on memory. Indeed, we aimed to extol the virtue of MPTs, compared to standard accuracy analyses, in making such assumptions more transparent.
